# Downregulation of cystathionine *β*‐synthase and cystathionine *γ*‐lyase expression stimulates inflammation in kidney ischemia–reperfusion injury

**DOI:** 10.14814/phy2.12251

**Published:** 2014-12-24

**Authors:** Pengqi Wang, Cara K. Isaak, Yaw L. Siow, Karmin O

**Affiliations:** 1 St. Boniface Hospital Research Centre, Winnipeg, Manitoba, Canada; 2 Department of Animal Science, University of Manitoba, Winnipeg, Manitoba, Canada; 3 Department of Physiology, University of Manitoba, Winnipeg, Manitoba, Canada; 4 Agriculture and Agri Food Canada, Winnipeg, Manitoba, Canada

**Keywords:** Cystathionine‐beta‐synthase, cystathionine‐gamma‐lyase, hydrogen sulfide, inflammation, ischemia–reperfusion, kidney

## Abstract

Inflammation plays a critical role in kidney ischemia–reperfusion injury but mechanisms of increased proinflammatory cytokine expression are not completely understood. Kidney has a high expression of cystathionine‐*β*‐synthase (CBS) and cystathionine‐*γ*‐lyase (CSE) that can synthesize hydrogen sulfide. CBE and CSE are also responsible for the synthesis of cysteine, an essential precursor for glutathione, an antioxidant. Reduced hydrogen sulfide and glutathione production is associated with multiple organ injury. Although pro‐ and anti‐inflammatory effects of hydrogen sulfide have been reported, its role in ischemia–reperfusion‐induced inflammation in the kidney has not been well addressed. The aim of this study was to investigate the effect of CBS and CSE‐mediated hydrogen sulfide and glutathione production on kidney inflammatory response and the mechanism involved. The left kidney of Sprague‐Dawley rat was subjected to 45‐min ischemia followed by reperfusion for 24 h. Ischemia–reperfusion caused a significant decrease in CBS and CSE mRNA and protein levels with a concomitant reduction of glutathione and hydrogen sulfide production in the kidney while the expression of proinflammatory cytokine expression (MCP‐1, IL‐6) was elevated. Hypoxia–reoxygenation of proximal tubular cells led to a decrease in CBS and CSE expression and an increase in proinflammatory cytokine expression. Supplementation of glutathione or hydrogen sulfide donor (NaHS) effectively attenuated cytokine expression in tubular cells. These results suggested that ischemia–reperfusion impaired CBS and CSE‐mediated glutathione and hydrogen sulfide production in the kidney, which augmented the expression of proinflammatory cytokines. Regulation of CBS and CSE expression may be therapeutically relevant in alleviating ischemia–reperfusion‐induced inflammation and improving kidney function.

## Abstract (precis)

Our study, for the first time, provides evidence that reduced CBS and CSE‐mediated hydrogen sulfide and glutathione production stimulate proinflammatory cytokine expression in the kidney upon ischemia–reperfusion injury. Restoration of CBS and CSE expression appears to be therapeutically relevant for AKI.

## Introduction

Kidney ischemia–reperfusion (I/R) is one of the common causes for acute kidney injury (AKI) which is associated with a high morbidity and mortality (Lameire et al. 2005; Hoste and Kellum 2007; Bonventre and Yang 2011; Bellomo et al. 2012). I/R injury occurs in many clinical conditions including renal transplantation and surgery. The pathogenesis of I/R‐induced kidney injury is multifaceted. Depletion of oxygen and nutrients, oxidative stress, inflammatory response, and induction of cell death contribute to kidney injury (Bonventre and Yang 2011; Sharfuddin and Molitoris 2011; Bellomo et al. 2012; Singh et al. 2012). I/R elicits inflammatory response characterized by increased expression of proinflammatory cytokines, which facilitates leukocyte recruitment into the kidney. The chain of events of I/R‐induced inflammatory response accelerates renal injury. Although inflammatory response is known to play a crucial role in kidney I/R injury, the mechanisms responsible for increased proinflammatory cytokine expression are not completely understood.

Inflammation is recognized as a major pathogenic process in kidney I/R injury (Bonventre and Zuk 2004; Thurman 2007; Kinsey et al. 2008; Sharfuddin and Molitoris 2011). I/R results in the generation of proinflammatory cytokines that play an important role in leukocyte recruitment and exacerbates kidney injury (Bonventre and Zuk 2004; Kinsey et al. 2008; Bonventre and Yang 2011). Monocyte chemoattractant protein‐1 (MCP‐1) is a potent chemotactic cytokine that stimulates monocyte recruitment to the kidney (Sung et al. 2002; Munshi et al. 2011). We and others have observed an increased expression of MCP‐1 and accumulation of leukocyte in rat kidney subjected to ischemia–reperfusion injury (Sung et al. 2002; Munshi et al. 2011). Attenuation of MCP‐1 expression significantly improved kidney function (Amann et al. 2003). Interleukin‐6 (IL‐6), one of the major inflammatory mediators, has been shown to have a strong correlation with the onset and severity of AKI (Kielar et al. 2005; Nechemia‐Arbely et al. 2008). Despite recent advances in AKI research, the mechanisms of kidney I/R‐induced inflammatory response remain to be further investigated.

Hydrogen sulfide (H_2_S) is a gasotransmitter that, at physiological levels, exerts beneficial effects through antioxidant and anti‐inflammatory action, as well as serving as a neurotransmitter (Abe and Kimura 1996; Elrod et al. 2007; Wallace et al. 2009; Gadalla and Snyder 2010; Wang 2012). Kidney is one of the major organs regulating endogenous H_2_S generation through cystathionine‐*β*‐synthase (CBS, EC 4.2.1.22) and cystathionine‐*γ*‐lyase (CSE, EC 4.4.1.1) (House et al. 1997; Prathapasinghe et al. 2007, 2008; Xu et al. 2009). These two enzymes catalyze H_2_S production through desulfhydration reactions (Fu et al. 2012). Both CBS and CSE are also responsible for the synthesis of cysteine through the transsulfuration pathway (Paul and Snyder 2012). Cysteine is an essential substrate for the biosynthesis of glutathione, a major endogenous antioxidant (Paul and Snyder 2012).

In a recent study, we have observed that ischemia followed by a short period of reperfusion (6 h) results in a significant decrease in hydrogen sulfide (H_2_S) production in rat kidney (Xu et al. 2009). Supplementation of exogenous H_2_S is shown to be renal protective in AKI and in chronic kidney disease (Xu et al. 2009; Lobb et al. 2012; Perna and Ingrosso 2012). In mice with CSE depletion, renal H_2_S production rate was markedly reduced (Bos et al. 2013). Generation of endogenous H_2_S by CSE or administration of H_2_S donor attenuates kidney I/R injury (Tripatara et al. 2008; Hunter et al. 2012). H_2_S has been implicated as a mediator exerting anti‐ and pro‐inflammatory effects (Whiteman and Winyard 2011; Rivers et al. 2012; Wang 2012; Chan and Wallace 2013). Although administration of exogenous H_2_S is shown to be cytoprotective in various organs of ischemia–reperfusion (Nicholson and Calvert 2010), the role of H_2_S in I/R‐induced inflammatory response in the kidney is not clear. The aim of this study was to investigate the effect of downregulation of CBS and CSE expression on endogenous H_2_S and glutathione production and its impact on kidney inflammatory response during ischemia followed by a longer period of reperfusion (24 h) injury.

## Materials and Methods

### Renal ischemia–reperfusion (I/R)

Kidney ischemia was induced in male Sprague–Dawley rats (250–300 g) by clamping the left renal pedicle for 45 min followed by reperfusion for 24 h (Sung et al. 2002; Prathapasinghe et al. 2007; Wang et al. 2013). In brief, rats were anesthetized by 3% isoflurane/oxygen gas prior to surgery. Surgery was performed when rats reach the stage 3 anesthesia. During the surgery, the 1–2% isoflurance/oxygen gas was maintained via inhalation. Rats were kept on a heat pad and the rectal temperature was maintained at 37°C throughout the experimental procedure. To prevent a decrease in body temperature, rats were placed in a warm incubator for 12 h after surgery. As a control, a sham‐operated group of rats were subjected to the same surgical procedure but without inducing I/R and were sacrificed at corresponding time points. A blood sample was collected and plasma was separated by centrifugation of blood at 3,000 *g* for 20 min at 4°C. Plasma creatinine level was measured by using a commercial assay kit (Genzyme diagnostics, Canada). Kidneys were harvested in ice‐cold potassium phosphate buffer. All procedures were performed in accordance with the Guide to the Care and Use of Experimental Animals published by the Canadian Council on Animal Care and approved by the University of Manitoba Protocol Management and Review Committee. All chemicals were obtained from Sigma‐Aldrich (St. Louis, MO) unless otherwise indicated.

### In vitro model of hypoxia–reoxygenation (HR) in cell culture

A hypoxia–reoxygenation (HR) model was used to simulate in vivo I/R injury. Human kidney cortex proximal tubular cells (HK‐2; CRL‐2190, American Type Culture Collection) were cultured in keratinocyte serum‐free medium supplied with human recombinant epidermal growth factor and bovine pituitary extract (Gibco/Invitrogen, Carlsbad, CA) at 37°C in a normal atmosphere of 95% air and 5% CO_2_. Hypoxia was induced in cells by oxygen–glucose deprivation (Wu et al. 2010). In brief, tubular cells were incubated for 2 h in a modified Krebs buffer (137 mmol/L NaCl, 3.8 mmol/L KCl, 0.49 mmol/L MgCl_2_, 0.9 mmol/L CaCl_2_, 4 mmol/L HEPES) supplemented with 10 mmol/L 2‐deoxyglucose, 20 mmol/L sodium lactate, 12 mmol/L KCl, and 1 mmol/L sodium dithionite (pH 6.2) in a hypoxia chamber (Billups‐Rothenberg, Inc., Del Mar, CA) containing 95% N_2_/5% CO_2_ at 37°C. Control cells were incubated in a modified Krebs buffer (pH 7.4) containing D‐glucose at 37°C in a normal atmosphere. After incubation for 2 h, the Krebs buffer was replaced with keratinocyte serum‐free medium and cells were cultured for another 24–48 h.

### Measurement of mRNA expression

Total RNAs were isolated from the kidney tissue with Trizol reagent (Invitrogen) according to the manufacturer's instruction. Total RNA (2 *μ*g) was converted to cDNA by reverse transcription. The mRNA expression of CBS, CSE, and cytokines was determined by a real‐time PCR analysis. The real‐time PCR reaction mixture contained 0.4 *μ*mol/L of 5′ and 3′ primers and 1 *μ*L of cDNA product in iQ‐SYBR green supermix reagent (Bio‐Rad, Mississauga, ON, Canada). The relative changes in mRNA expression were determined by the fold change analysis. The primers (Invitrogen) used in this study are listed in Table 1.

**Table 1.** Gene primer sequences used for real‐time PCR.

Target gene	Forward Primer(5′‐3′)	Reverse Primer(5′‐3′)
Human		
CBS	GCAGATCCAGTACCACAGCA	CTCCGGACTTCACTTCTGGT
CSE	CAAGGTTTCCTGCCACACTT	GCTATATTCAAAACCCGAGTGC
IL‐6	AGGAGACTTGCCTGGTGAAA	GTCAGGGGTGGTTATTGCAT
MCP‐1	CCCAAAGAAGCTGTGATCTTCA	GTGTCTGGGGAAAGCTAGGG
GAPDH	GAGCGAGATCCCTCCAAAAT	GGCTGTTGTCATACTTCTCATGG
Rat		
CBS	TCGTGATGCCTGAGAAGATG	TTGGGGATTTCGTTCTTCAG
CSE	GTATGGAGGCACCAACAGGT	GTTGGGTTTGTGGGTGTTTC
IL‐6	CCGGAGAGGAGACTTCACAG	ACAGTGCATCATCGCTGTTC
MCP‐1	CAGAAACCAGCCAACTCTCA	AGACAGCACGTGGATGCTAC
*β*‐actin	ACAACCTTCTTGCAGCTCCTC	GACCCATACCCACCATCACA

### Western immunoblotting analysis of CBS and CSE proteins, measurement of cytokines

The protein levels of CBS and CSE in the kidney were measured by Western immunoblotting analysis. In brief, kidney proteins (20 *μ*g) were separated by electrophoresis in 10% SDS polyacrylamide gels. Proteins in the gel were transferred to a nitrocellulose membrane. The membrane was probed with (Abe and Kimura 1996) mouse anti‐CBS monoclonal (1:3,000; Abnova Corporation, Taipei, Taiwan) or rabbit anti‐CSE monoclonal antibodies (1:3,000; GeneTex, Irvine, CA) for rat proteins. HRP‐conjugated anti‐mouse or anti‐rabbit IgG antibodies (Cell Signaling Technology, Danvers, MA) were used as the secondary antibodies (1:5,000). The corresponding protein bands were visualized using enhanced chemiluminescence reagents and analyzed with a gel documentation system (Bio‐Rad Gel Doc1000). To confirm the equal loading of proteins for each sample, the same membranes were reprobed with mouse anti‐*β*‐actin monoclonal antibodies (1:5,000, Cell Signaling Technology). Proinflammatory factors (MCP‐1, IL‐6) in the kidney and plasma as well as plasma neutrophil gelatinase‐associated lipocalin (NGAL) were measured using the MesoScale Discovery electrochemiluminescence platform (Rockville, MD).

### Measurement of H_2_S production and glutathione levels in the kidney

H_2_S production was measured based on a method described by Stipanuk and Beck (Stipanuk and Beck 1982). Kidney tissue was homogenized in 50 mmol/L potassium phosphate buffer (pH 6.9) followed by centrifugation at 15,000 *g* for 30 min at 4°C. The supernatant was collected and H_2_S production was measured in a reaction mixture containing 0.3 mL supernatant, 10 mmol/L l‐cysteine, 10 mmol/L DL‐Hcy, 2 mmol/L pyridoxal‐5′‐phosphate, and 0.05 mmol/L S‐adenosylmethionine and prepared in 100 mmol/L potassium phosphate buffer (pH 7.4). The reaction was carried out in an Erlenmeyer flask that was fitted with a septum stopper and contained a plastic center well. A folded filter paper was soaked in 0.5 ml mixture of 1% zinc acetate and 12% NaOH. The tube was placed in the flask and the flask was blown with N_2_. The flask was immediately covered and incubated in a water bath for 30 min at 37°C. The reaction was stopped with the injection of 30% trichloroacetic acid into the flask. The flask was incubated for an additional 60 min at 37°C. The filter paper was removed and transferred to a test tube containing 3.5 mL water to which 0.4 mL of 20 mmol/L *N*,*N*,dimethyl‐*p*‐phenylenediamine sulfate dissolved in 7.2 M HCl and 0.4 mL of 30 mmol/L FeCl_3_ dissolved in 1.2 M HCl were added. The reaction was allowed to proceed for 10 min in the dark and the absorbance of the resulting solution was measured at 670 nm. Sodium hydrosulfide hydrate was used as standard (Xu et al. 2009; Hwang et al. 2013). Total glutathione was measured in liver tissue and cell lysates as previously reported (Rahman et al. 2006).

### Statistical analysis

Results were analyzed using one‐way ANOVA followed by Newman–Keuls test. Data were presented as the means ± SEM. The level of statistical significance was determined when a *P* value was less than 0.05.

## Results

### Effect of ischemia–reperfusion on kidney function, H_2_S production, and glutathione level

**Figure 1.**
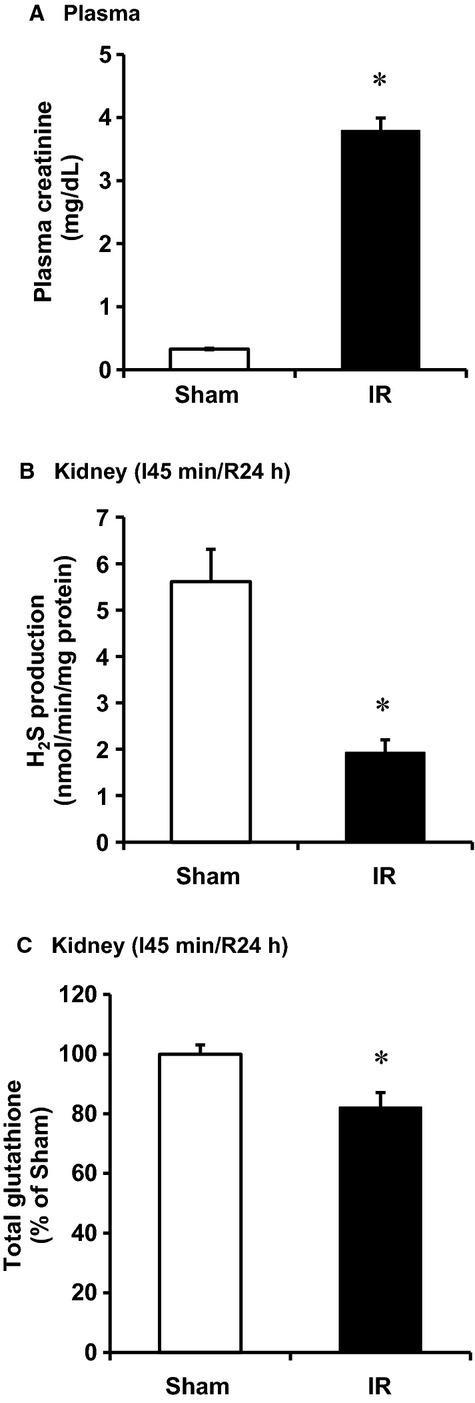
Effect of ischemia–reperfusion on plasma creatinine level, kidney H_2_S production, and glutathione level in rats. The left kidney of rats was subjected 45‐min ischemia followed by 24‐h reperfusion (I/R). As a control, rats were subjected to a sham operation but without inducing ischemia–reperfusion. (A) Plasma creatinine levels were determined. (B) H_2_S production and (C) glutathione level were measured in the kidney tissue. Results are expressed as mean ± SEM (*n* = 5 for each group). *P < 0.05 when compared with the value obtained from the sham‐operated group.

The induction of kidney ischemia (45 min) followed by reperfusion for 24 h resulted in a marked elevation of plasma creatinine (Fig. 1A), indicating that kidney function was impaired. Upon ischemia–reperfusion, the H_2_S production in the kidney tissue was significantly decreased (Fig. 1B). Total glutathione level was significantly lower in ischemia‐reperfused kidneys than that in the sham‐operated group (Fig. 1C).

### Effect of ischemia–reperfusion on the expression of CBS, CSE, and proinflammatory cytokines in the kidney

**Figure 2.**
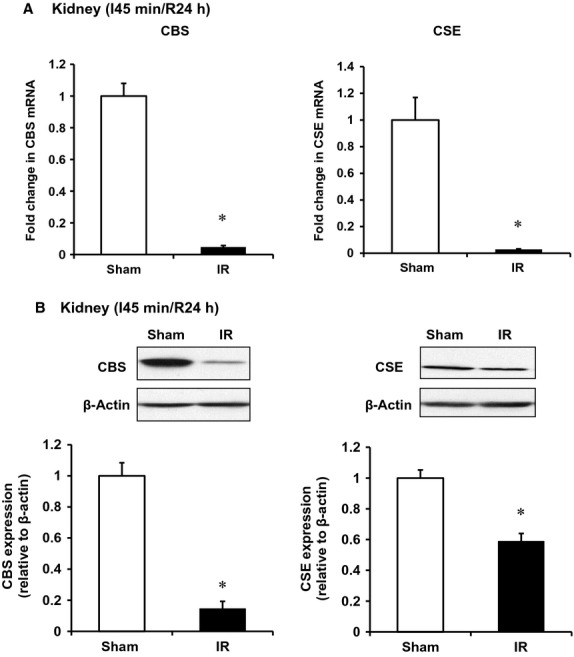
Effect of ischemia–reperfusion on CBS and CSE expression in the kidney. The left kidney of rats was subjected 45‐min ischemia followed by 24‐h reperfusion (I/R). As a control, rats were subjected to a sham operation but without inducing I/R. The mRNA (A) and protein (B) of CBS and CSE in the kidney tissue were determined by a real‐time PCR and Western immunoblotting analysis, respectively. Results are expressed as mean ± SEM (*n* = 5 for each group). *P < 0.05 when compared with the value obtained from the sham‐operated group.

**Figure 3.**
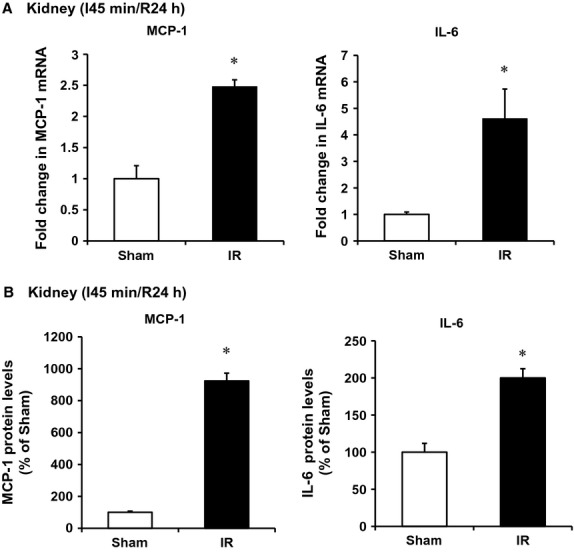
Effect of ischemia–reperfusion on cytokine expression in the kidney. The left kidney of rats was subjected 45‐min ischemia followed by 24‐h reperfusion (I/R). As a control, rats were subjected to a sham operation but without inducing ischemia–reperfusion. The mRNA (A) levels and protein (B) of cytokines (MCP‐1, IL‐6) were measured in the kidney tissue. Results are expressed as mean ± SEM (*n* = 5 for each group). *P < 0.05 when compared with the value obtained from the sham‐operated group.

**Figure 4.**
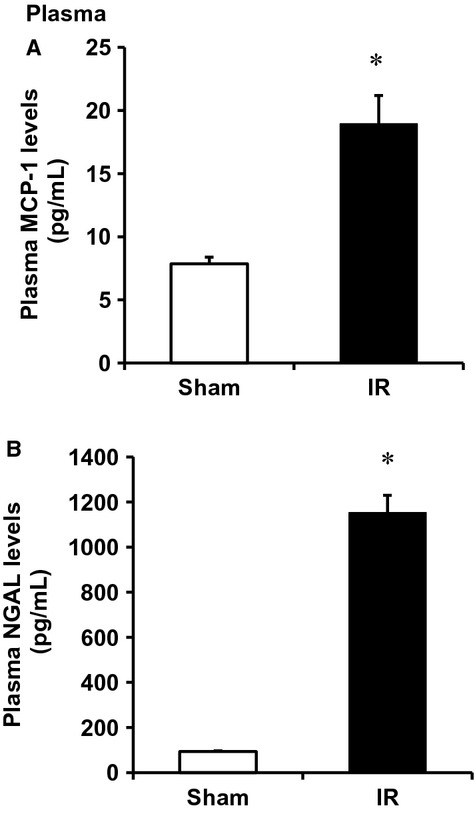
Effect of ischemia–reperfusion on plasma MCP‐1 and NGAL levels. The left kidney of rats was subjected 45‐min ischemia followed by 24‐h reperfusion (I/R). As a control, rats were subjected to a sham operation but without inducing ischemia–reperfusion. The MCP‐1 (A) and NGAL (B) levels were measured in plasma. Results are expressed as mean ± SEM (*n* = 5 for each group). *P<0.05 when compared with the value obtained from the sham‐operated group.

To investigate whether a decrease in H_2_S levels was due to downregulation of CBS and CSE expression in the kidney, CBS, and CSE mRNA was measured by a real‐time PCR analysis. The levels of CBS and CSE mRNA were significantly lower in I/R kidneys than that in the sham‐operated group (Fig. 2A). In accordance, the protein levels of these two enzymes were significantly decreased in those kidneys (Fig. 2B). We then examined the effect of I/R on inflammatory response in the kidney. There was a significant increase in proinflammatory cytokines (MCP‐1, IL‐6) mRNA and protein levels in kidneys subjected to I/R (Fig. 3). The level of MCP‐1 was also significantly elevated in the plasma of rats subjected to kidney I/R (Fig. 4A). Neutrophil gelatinase‐associated lipocalin (NGAL) is a small (25‐kd) protein that belongs to the lipocalin protein family. NGAL is produced by epithelial cells and neutrophils in response to tubular epithelial damage (Sharfuddin and Molitoris 2011). Plasma NGAL has been proposed as a marker of tubular damage in AKI (Sodha et al. 2009; Singh et al. 2012). The level of plasma NGAL was significantly elevated (Fig. 4B), indicating tubular damage in I/R‐induced kidney injury.

### Role of the transsulfuration pathway in ischemia–reperfusion‐stimulated cytokine expression in the kidney and in tubular cells

**Figure 5.**
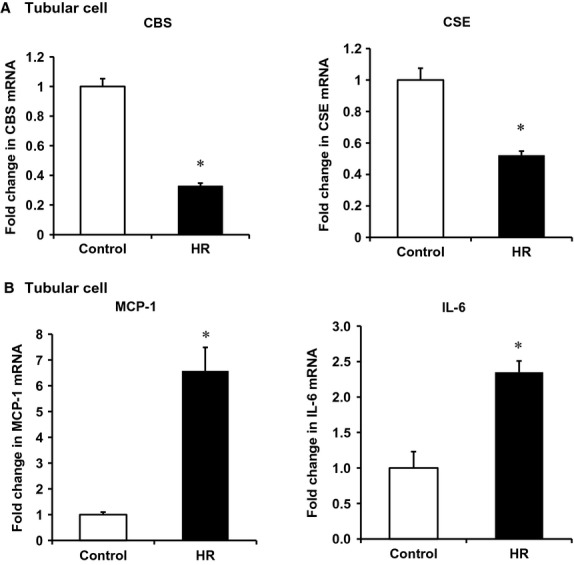
Effect of hypoxia–reoxygenation on the expression of CBS, CSE and cytokines in tubular cells. Human proximal tubular cells were subjected to 2‐h hypoxia followed by 24‐h reoxygenation (HR). Cells cultured in the regular medium without being subjected to HR were used as a control. A real‐time PCR analysis was used to measure the mRNA of CBS and CSE (A) and cytokines (MCP‐1, IL‐6) (B). Results are expressed as mean ± SEM (*n* = 5 for each group). *P < 0.05 when compared with the value obtained from control cells.

**Figure 6.**
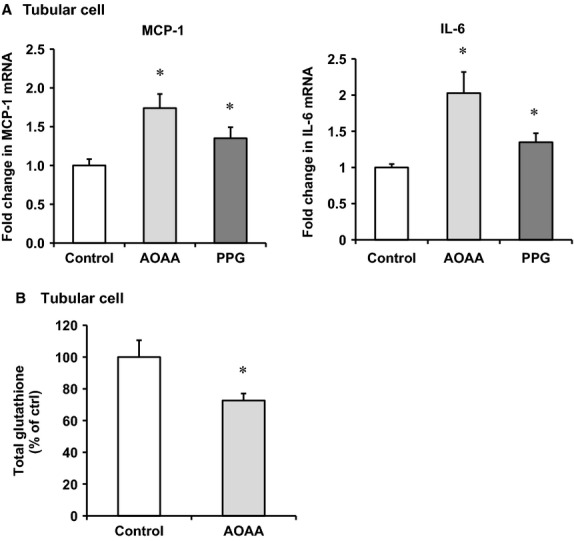
Effect of CBS and CSE inhibitors on proinflammatory cytokine expression and glutathione levels in tubular cells. Human proximal tubular cells were incubated in the absence (control) or presence of a CBS inhibitor aminooxyacetic acid (AOAA, 0.2 mmol/L) or a CSE inhibitor DL‐propargylglycine (PAG, 0.5 mmol/L). The mRNA of MCP‐1 and IL‐6 was measured by a real‐time PCR analysis (A). Total intracellular glutathione levels were measured (B). Results are expressed as mean ± SEM (*n* = 5 for each group). *P < 0.05 when compared with the value obtained from control cells.

**Figure 7.**
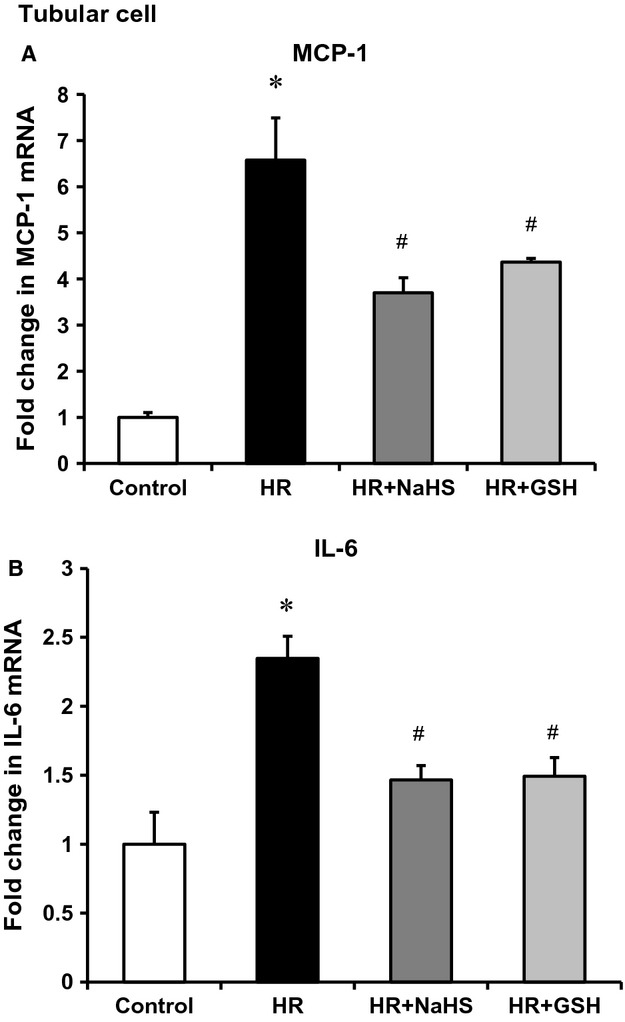
Effects of H_2_S donor and glutathione on proinflammatory cytokine expression in tubular cells. Human proximal tubular cells were subjected to hypoxia 2 h followed by 48‐h reoxygenation in the absence (HR) or presence of NaHS (10 μmol/L) or glutathione (GSH 0.01 mmol/L). Cells cultured in a regular medium without being subjected to HR were used as a control. The mRNA levels of MCP‐1 (A) and IL‐6 (B) were measured by a real‐time PCR analysis. Results are expressed as mean ± SEM (*n* = 5 for each group). *P < 0.05 when compared with the value obtained from control cells. ^#^*P *< 0.05 when compared with the value obtained from cells subjected to hypoxia–reoxygenation.

To investigate whether downregulation of CBS and CSE expression contributed to increased cytokine expression, experiments were conducted in human proximal tubular cells, the type of cells in the kidney where CBS and CSE are highly expressed. Tubular cells were subjected to hypoxia followed by reoxygenation with regular culture medium (reperfusion). There was a significant reduction of CBS and CSE mRNA levels in cells subjected to 2 h of hypoxia followed by reoxygenation with a regular culture medium for 24 h (Fig. 5A) while the levels of proinflammatory cytokines MCP‐1 and IL‐6 were significantly elevated (Fig. 5B). Next, tubular cells were incubated with a CBS inhibitor aminooxyacetic acid (AOAA) or a CSE inhibitor DL‐propargylglycine (PAG). Inhibition of CBS and CSE led to an elevation of MCP‐1 and IL‐6 mRNA in these cells (Fig. 6A). Inhibition of rate‐limiting enzyme CBS in the transsulfuration pathway caused a significant reduction of intracellular glutathione levels in tubular cells (Fig. 6B). To further investigate whether a reduction of CBS and CSE‐mediated H_2_S and glutathione production played a crucial role in proinflammatory cytokine expression, hydrogen sulfide donor sodium hydrosulfide (NaHS) and glutathione (GSH) were added to the culture medium prior to induction of hypoxia–reoxygenation. Addition of NaHS or GSH attenuated hypoxia–reoxygenation‐induced MCP‐1 and IL‐6 expression in tubular cells (Fig. 7).

## Discussion

Acute inflammatory response is a hallmark of I/R‐induced kidney injury. Uncontrolled inflammatory response can exacerbate I/R injury. However, the mechanisms responsible for aberrant inflammatory response are not completely understood. This study for the first time demonstrates that reduced CBS and CSE expression contributes to inflammatory response in the kidney upon 45‐min ischemia followed by 24‐h reperfusion. Inhibition of CBS and CSE leads to reduced glutathione and H_2_S production in the kidney and in proximal tubular cells, which correlates with increased expression of proinflammatory cytokines. Supplementation of glutathione or H_2_S donor effectively attenuates the expression of proinflammatory cytokines in tubular cells.

Both CBS and CSE in the transsulfuration pathway are highly expressed in the kidney. These two enzymes are mainly located in the proximal tubule segments which are more susceptible to I/R injury (Beltowski 2010). In this study, tubular damage persisted 24 h after ischemia insult as indicated by a significantly elevated plasma NGAL level. The expression of CBS and CSE was significantly reduced in I/R kidney. Cysteine lies downstream of the transsulfuration pathway and serves as an essential substrate for glutathione biosynthesis. A decrease in CBS and CSE expression in the transsulfuration pathway could lead to a reduction of cysteine level and subsequently limit glutathione generation. In this study, I/R caused a significant reduction in glutathione levels in the kidney. Such an inhibitory effect on glutathione levels was also observed in tubular cells subjected to hypoxia–reoxygenation. Inhibition of the rate‐limiting enzyme CBS also led to a decrease in intracellular glutathione levels in tubular cells. These results suggested that reduction of CBS and CSE expression in the transsulfuration pathway was responsible for glutathione depletion in the kidney upon I/R injury. Glutathione is a major endogenous nonenzymatic antioxidant that has anti‐inflammatory property through the regulation of redox‐sensitive signaling pathways. Acute inflammatory response is recognized as one of the major factors of kidney I/R injury, which is characterized by increased expression of proinflammatory cytokines and recruitment of leukocytes. In this study, supplementation of glutathione to tubular cells effectively attenuated the expression of proinflammatory cytokines. Such beneficial effect of glutathione might be mediated via its antioxidant action.

**Figure 8.**
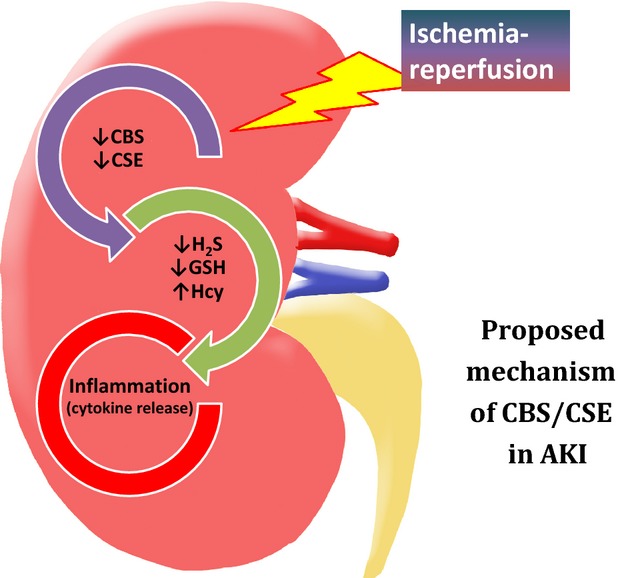
Role of CBS and CSE mediated H_2_S and glutathione production in inflammatory response in kidney ischemia–reperfusion injury. The role of the CBS/CSE system in proinflammatory cytokine expression is summarized in the context of kidney ischemia–reperfusion (I/R)‐induced acute kidney injury (AKI).

Aside from their roles in the transsulfuration pathway, CBS and CSE are also a major source of H_2_S production through desulfhydration reactions (Xu et al. 2009). In accordance with a reduction in CBS and CSE expression, there was a marked decrease in H_2_S production in the kidney subjected to 45‐min ischemia and 24‐h reperfusion. H_2_S has been shown to have protective effects against I/R injury in various organs including heart, brain, kidney, and liver (Calvert et al. 2009; Kang et al. 2009; Xu et al. 2009; Gheibi et al. 2014). Anti‐ and pro‐inflammatory effects of endogenous H_2_S or exogenous H_2_S donor are reported in various animal models (Whiteman and Winyard 2011). The mechanisms by which H_2_S exerts inflammatory response are complex including attenuation of nuclear factor‐*κ*B (NF‐*κ*B) activation, reduction of proinflammatory factor expression, and inhibition of leukocyte adhesion (Zhang et al. 2008; Dongo et al. 2011; Whiteman and Winyard 2011). For example, in a myocardial I/R injury porcine model, administration of H_2_S donor (NaHS) prior to reperfusion limited inflammatory response and attenuated myocardial injury in Yorkshire pigs (Sodha et al. 2009). In a cerebral I/R injury rat model, administration of NaHS exerted a protective effect against severe cerebral injury induced by a global I/R through attenuation of oxidative stress, inflammation, and apoptosis in the brain tissue (Yin et al. 2013). In mouse mesangial cells, increased endogenous H_2_S generation or supplementation of exogenous H_2_S partially inhibited Hcy‐induced expression of proinflammatory cytokines (Sen et al. 2011). We previously reported acute inhibition of CBS‐mediated H_2_S production in I/R kidney 6 h after the onset of reperfusion (Xu et al. 2009). Results obtained from this study indicated that the expression of both CBS and CSE was reduced in the kidney subjected to 45‐min ischemia followed by 24‐h reperfusion. Although H_2_S has been implicated in the regulation of inflammatory response, its role in the expression of proinflammatory cytokines in AKI has not been well defined. Our recent study has demonstrated that administration of H_2_S donor (NaHS) can restore I/R‐impaired kidney function (Xu et al. 2009). This study provided several lines of evidence indicating that changes in CBS and CSE‐mediated H_2_S production had a profound effect on I/R‐induced proinflammatory cytokine expression. First, low CBS and CSE expression along with decreased H_2_S production was inversely associated with I/R‐induced expression of proinflammatory cytokines (i.e., MCP‐1, IL‐6) in the kidney. Second, the expression of CBS and CSE was decreased in proximal tubular cells subjected to hypoxia–reoxygenation, while the expression of proinflammatory cytokines such as IL‐6 and MCP‐1 was elevated in these cells. Exogenous H_2_S donor effectively attenuated I/R‐induced cytokine expression in tubular cells. Third, inhibition of H_2_S production by CBS and CSE inhibitors also led to an increase in proinflammatory cytokine expression in tubular cells. Taken together, these results suggested that reduced endogenous H_2_S generation due to downregulation of CBS and CSE contributed to increased expression of proinflammatory cytokines in the kidney upon I/R injury. Given the anti‐inflammatory property of H_2_S, its deficiency could potentially exacerbate I/R injury in the kidney. Our results indicated that CBS and CSE played an important role in regulating H_2_S generation in the kidney. Reduction in CBS and CSE‐mediated H_2_S production during I/R contributed to inflammatory response in the kidney (Fig. 8). Proper restoration of endogenous H_2_S production and/or administration of exogenous H_2_S donor may represent one of the therapeutic targets in I/R‐mediated tissue injury.

In conclusion, this study clearly demonstrates that I/R injury decreases CBS and CSE expression leading to a significant reduction of endogenous H_2_S and glutathione production in the kidney. Reduction of H_2_S and glutathione contributes to increased proinflammatory cytokine expression in the kidney and in tubular cells upon I/R or hypoxia–reoxygenation insult. Regulation of endogenous H_2_S and glutathione homeostasis plays an important role in anti‐inflammatory response in the kidney. Our results, for the first time, suggest that downregulation of CBS and CSE mediated H_2_S and glutathione production contributes to the pathogenesis of I/R‐induced kidney injury. Restoration of CBS and CSE expression appears to be therapeutically relevant for AKI.

## Conflict of Interest

No conflicts of interest, financial or otherwise, are declared by the authors.

## References

[b1] Abe K. Kimura H. 1996 The possible role of hydrogen sulfide as an endogenous neuromodulator. J. Neurosci.; 16:1066-1071. 8558235 10.1523/JNEUROSCI.16-03-01066.1996 PMC6578817

[b2] Amann B. Tinzmann R. Angelkort B. 2003 ACE inhibitors improve diabetic nephropathy through suppression of renal MCP‐1. Diabetes Care; 26:2421-2425. 12882873 10.2337/diacare.26.8.2421

[b3] Bellomo R. Kellum J. A. Ronco C. 2012 Acute kidney injury. Lancet; 380:756-766. 22617274 10.1016/S0140-6736(11)61454-2

[b4] Beltowski J. 2010 Hypoxia in the renal medulla: implications for hydrogen sulfide signaling. J. Pharmacol. Exp. Ther.; 334:358-363. 20427475 10.1124/jpet.110.166637

[b5] Bonventre J. V. Yang L. 2011 Cellular pathophysiology of ischemic acute kidney injury. J. Clin. Invest.; 121:4210-4221. 22045571 10.1172/JCI45161 PMC3204829

[b6] Bonventre J. V. Zuk A. 2004 Ischemic acute renal failure: an inflammatory disease? Kidney Int.; 66:480-485. 15253693 10.1111/j.1523-1755.2004.761_2.x

[b7] Bos E. M. Wang R. Snijder P. M. Boersema M. Damman J. Fu M. 2013 Cystathionine gamma‐lyase protects against renal ischemia/reperfusion by modulating oxidative stress. J. Am. Soc. Nephrol.; 24:759-770. 23449534 10.1681/ASN.2012030268 PMC3636788

[b8] Calvert J. W. Jha S. Gundewar S. Elrod J. W. Ramachandran A. Pattillo C. B. 2009 Hydrogen sulfide mediates cardioprotection through Nrf2 signaling. Circ. Res.; 105:365-374. 19608979 10.1161/CIRCRESAHA.109.199919 PMC2735849

[b9] Chan M. V. Wallace J. L. 2013 Hydrogen sulfide‐based therapeutics and gastrointestinal diseases: translating physiology to treatments. Am. J. Physiol. Gastrointest. Liver Physiol.; 305:G467-G473. 23868410 10.1152/ajpgi.00169.2013

[b10] Dongo E. Hornyak I. Benko Z. Kiss L. 2011 The cardioprotective potential of hydrogen sulfide in myocardial ischemia/reperfusion injury (review). Acta Physiol. Hung.; 98:369-381. 22173019 10.1556/APhysiol.98.2011.4.1

[b11] Elrod J. W. Calvert J. W. Morrison J. Doeller J. E. Kraus D. W. Tao L. 2007 Hydrogen sulfide attenuates myocardial ischemia‐reperfusion injury by preservation of mitochondrial function. Proc. Natl. Acad. Sci. USA; 104:15560-15565. 17878306 10.1073/pnas.0705891104 PMC2000503

[b12] Fu M. Zhang W. Wu L. Yang G. Li H. Wang R. 2012 Hydrogen sulfide (H2S) metabolism in mitochondria and its regulatory role in energy production. Proc. Natl. Acad. Sci. USA; 109:2943-2948. 22323590 10.1073/pnas.1115634109 PMC3287003

[b13] Gadalla M. M. Snyder S. H. 2010 Hydrogen sulfide as a gasotransmitter. J. Neurochem.; 113:14-26. 20067586 10.1111/j.1471-4159.2010.06580.x PMC2965526

[b14] Gheibi S. Aboutaleb N. Khaksari M. Kalalian‐Moghaddam H. Vakili A. Asadi Y. 2014 Hydrogen sulfide protects the brain against ischemic reperfusion injury in a transient model of focal cerebral ischemia. J. Mol. Neurosci.; 54:264-270. 24643521 10.1007/s12031-014-0284-9

[b15] Hoste E. A. Kellum J. A. 2007 Incidence, classification, and outcomes of acute kidney injury. Contrib. Nephrol.; 156:32-38. 17464113 10.1159/000102013

[b16] House J. D. Brosnan M. E. Brosnan J. T. 1997 Characterization of homocysteine metabolism in the rat kidney. Biochem. J.; 328Pt 1:287-292. 9359866 10.1042/bj3280287 PMC1218919

[b17] Hunter J. P. Hosgood S. A. Patel M. Rose R. Read K. Nicholson M. L. 2012 Effects of hydrogen sulphide in an experimental model of renal ischaemia‐reperfusion injury. Br. J. Surg.; 99:1665-1671. 23132416 10.1002/bjs.8956

[b18] Hwang S. Y. Sarna L. K. Siow Y. L. O K. 2013 High‐fat diet stimulates hepatic cystathionine beta‐synthase and cystathionine gamma‐lyase expression. Can. J. Physiol. Pharmacol.; 91:913-919. 24117258 10.1139/cjpp-2013-0106

[b19] Kang K. Zhao M. Jiang H. Tan G. Pan S. Sun X. 2009 Role of hydrogen sulfide in hepatic ischemia‐reperfusion‐induced injury in rats. Liver Transpl.; 15:1306-1314. 19790158 10.1002/lt.21810

[b20] Kielar M. L. John R. Bennett M. Richardson J. A. Shelton J. M. Chen L. 2005 Maladaptive role of IL‐6 in ischemic acute renal failure. J. Am. Soc. Nephrol.; 16:3315-3325. 16192425 10.1681/ASN.2003090757

[b21] Kinsey G. R. Li L. Okusa M. D. 2008 Inflammation in acute kidney injury. Nephron Exp. Nephrol.; 109:e102-e107. 18802372 10.1159/000142934 PMC2614446

[b22] Lameire N. Van Biesen W. Vanholder R. 2005 Acute renal failure. Lancet; 365:417-430. 15680458 10.1016/S0140-6736(05)17831-3

[b23] Lobb I. Mok A. Lan Z. Liu W. Garcia B. Sener A. 2012 Supplemental hydrogen sulphide protects transplant kidney function and prolongs recipient survival after prolonged cold ischaemia‐reperfusion injury by mitigating renal graft apoptosis and inflammation. BJU Int.; 110:E1187-E1195. 23157304 10.1111/j.1464-410X.2012.11526.x

[b24] Munshi R. Johnson A. Siew E. D. Ikizler T. A. Ware L. B. Wurfel M. M. 2011 MCP‐1 gene activation marks acute kidney injury. J. Am. Soc. Nephrol.; 22:165-175. 21071523 10.1681/ASN.2010060641 PMC3014045

[b25] Nechemia‐Arbely Y. Barkan D. Pizov G. Shriki A. Rose‐John S. Galun E. 2008 IL‐6/IL‐6R axis plays a critical role in acute kidney injury. J. Am. Soc. Nephrol.; 19:1106-1115. 18337485 10.1681/ASN.2007070744 PMC2396933

[b26] Nicholson C. K. Calvert J. W. 2010 Hydrogen sulfide and ischemia‐reperfusion injury. Pharmacol. Res.; 62:289-297. 20542117 10.1016/j.phrs.2010.06.002 PMC2917489

[b27] Paul B. D. Snyder S. H. 2012 H(2)S signalling through protein sulfhydration and beyond. Nat. Rev. Mol. Cell Biol.; 13:499-507. 22781905 10.1038/nrm3391

[b28] Perna A. F. Ingrosso D. 2012 Low hydrogen sulphide and chronic kidney disease: a dangerous liaison. Nephrol. Dial. Transplant.; 27:486-493. 22323660 10.1093/ndt/gfr737

[b29] Prathapasinghe G. A. Siow Y. L. O K. 2007 Detrimental role of homocysteine in renal ischemia‐reperfusion injury. Am. J. Physiol. Renal. Physiol.; 292:F1354-F1363. 17264313 10.1152/ajprenal.00301.2006

[b30] Prathapasinghe G. A. Siow Y. L. Xu Z. O K. 2008 Inhibition of cystathionine‐beta‐synthase activity during renal ischemia‐reperfusion: role of pH and nitric oxide. Am. J. Physiol. Renal. Physiol.; 295:F912-F922. 18701635 10.1152/ajprenal.00040.2008

[b31] Rahman I. Kode A. Biswas S. K. 2006 Assay for quantitative determination of glutathione and glutathione disulfide levels using enzymatic recycling method. Nat. Protoc.; 1:3159-3165. 17406579 10.1038/nprot.2006.378

[b32] Rivers J. R. Badiei A. Bhatia M. 2012 Hydrogen sulfide as a therapeutic target for inflammation. Expert Opin. Ther. Targets; 16:439-449. 22448627 10.1517/14728222.2012.673591

[b33] Sen U. Givvimani S. Abe O. A. Lederer E. D. Tyagi S. C. 2011 Cystathionine beta‐synthase and cystathionine gamma‐lyase double gene transfer ameliorate homocysteine‐mediated mesangial inflammation through hydrogen sulfide generation. Am. J. Physiol. Cell Physiol.; 300:C155-C163. 20943958 10.1152/ajpcell.00143.2010 PMC3023186

[b34] Sharfuddin A. A. Molitoris B. A. 2011 Pathophysiology of ischemic acute kidney injury. Nat. Rev. Nephrol.; 7:189-200. 21364518 10.1038/nrneph.2011.16

[b35] Singh A. P. Junemann A. Muthuraman A. Jaggi A. S. Singh N. Grover K. 2012 Animal models of acute renal failure. Pharmacol. Rep.; 64:31-44. 22580518 10.1016/s1734-1140(12)70728-4

[b36] Sodha N. R. Clements R. T. Feng J. Liu Y. Bianchi C. Horvath E. M. 2009 Hydrogen sulfide therapy attenuates the inflammatory response in a porcine model of myocardial ischemia/reperfusion injury. J. Thorac. Cardiovasc. Surg.; 138:977-984. 19660398 10.1016/j.jtcvs.2008.08.074 PMC2758694

[b37] Stipanuk M. H. Beck P. W. 1982 Characterization of the enzymic capacity for cysteine desulphhydration in liver and kidney of the rat. Biochem. J.; 206:267-277. 7150244 10.1042/bj2060267 PMC1158582

[b38] Sung F. L. Zhu T. Y. Au‐Yeung K. K. Siow Y. L. O K. 2002 Enhanced MCP‐1 expression during ischemia/reperfusion injury is mediated by oxidative stress and NF‐kappaB. Kidney Int.; 62:1160-1170. 12234286 10.1111/j.1523-1755.2002.kid577.x

[b39] Thurman J. M. 2007 Triggers of inflammation after renal ischemia/reperfusion. Clin. Immunol.; 123:7-13. 17064966 10.1016/j.clim.2006.09.008 PMC1888143

[b40] Tripatara P. Patel N. S. Collino M. Gallicchio M. Kieswich J. Castiglia S. 2008 Generation of endogenous hydrogen sulfide by cystathionine gamma‐lyase limits renal ischemia/reperfusion injury and dysfunction. Lab. Invest.; 88:1038-1048. 18679378 10.1038/labinvest.2008.73

[b41] Wallace J. L. Vong L. McKnight W. Dicay M. Martin G. R. 2009 Endogenous and exogenous hydrogen sulfide promotes resolution of colitis in rats. Gastroenterology; 137:569-578. 19375422 10.1053/j.gastro.2009.04.012

[b42] Wang R. 2012 Physiological implications of hydrogen sulfide: a whiff exploration that blossomed. Physiol. Rev.; 92:791-896. 22535897 10.1152/physrev.00017.2011

[b43] Wang P. Zhu Q. Wu N. Siow Y. L. Aukema H. O K. 2013 Tyrosol attenuates ischemia‐reperfusion‐induced kidney injury via inhibition of inducible nitric oxide synthase. J. Agric. Food Chem.; 61:3669-3675. 23566115 10.1021/jf400227u

[b44] Whiteman M. Winyard P. G. 2011 Hydrogen sulfide and inflammation: the good, the bad, the ugly and the promising. Expert Rev. Clin. Pharmacol.; 4:13-32. 22115346 10.1586/ecp.10.134

[b45] Wu N. Siow Y. L. O K. 2010 Ischemia/reperfusion reduces transcription factor Sp1‐mediated cystathionine beta‐synthase expression in the kidney. J. Biol. Chem.; 285:18225-18233. 20392694 10.1074/jbc.M110.132142 PMC2881747

[b46] Xu Z. Prathapasinghe G. Wu N. Hwang S. Y. Siow Y. L. O K. 2009 Ischemia‐reperfusion reduces cystathionine‐beta‐synthase‐mediated hydrogen sulfide generation in the kidney. Am. J. Physiol. Renal. Physiol.; 297:F27-F35. 19439522 10.1152/ajprenal.00096.2009

[b47] Yin J. Tu C. Zhao J. Ou D. Chen G. Liu Y. 2013 Exogenous hydrogen sulfide protects against global cerebral ischemia/reperfusion injury via its anti‐oxidative, anti‐inflammatory and anti‐apoptotic effects in rats. Brain Res.; 1491:188-196. 23123706 10.1016/j.brainres.2012.10.046

[b48] Zhang H. Moochhala S. M. Bhatia M. 2008 Endogenous hydrogen sulfide regulates inflammatory response by activating the ERK pathway in polymicrobial sepsis. J. Immunol.; 181:4320-4331. 18768890 10.4049/jimmunol.181.6.4320

